# Characterization of the complete chloroplast genome of *Centaurea maculosa* (Asteraceae)

**DOI:** 10.1080/23802359.2019.1687342

**Published:** 2019-11-12

**Authors:** Kyu Tae Park, Lajin Park, Joo-Hwan Kim, Seonjoo Park

**Affiliations:** a Department of Life Science, Yeungnam University, Gyeongsan, Korea;; b Macrogen Inc., Seoul, Korea;; c Department of Life Science, Gachon University, Seongnam-si, Korea

**Keywords:** *Centaurea maculosa*, complete chloroplast genome, Asteraceae, invasive species

## Abstract

The complete chloroplast genome of *Centaurea maculosa* (Asteraceae) was presented in this article. The genome size is 152,518 bp in length, with 43.1% of GC content. It consists of a large single-copy (LSC) region (83,959 bp) and a small single-copy region (SSC) (18,487 bp) which were separated by two inverted repeat (IRs) regions (25,218 bp). The complete chloroplast genome contains 111 unique genes, including 80 coding genes, 4 rRNA genes, and 27 tRNA genes.

*Centaurea maculosa* Lam. (Asteraceae) is the Eurasian forb, known as an invasive plant in North America. It was introduced to the Pacific North West (US and Canada) in the 1890s and is spreading rapidly on semi-arid grasslands (Lacey et al. 1995). Root exudes chemical compounds (herbicide) which inhibits the native species. Moreover, without its native enemies, it could be more competitive than native species (Callaway et al. 2001). This work aims to contribute to provide genetic information of *C. maculosa* as invasive species.

*Centaurea maculosa* was collected from Occoquan Bay National Wild Life Refuge in US (N38°38′38″, W77°14′13″) and the total DNA was extracted using the DNeasy plant Mini Kit (Qiagen, Carlsbad, CA) and its DNA (YNUHD19039) was stored at Yeungnam University Herbarium (YNUH). The complete chloroplast genome of *C. maculosa* was sequenced by HiSeq2000 sequencer of Illumina (San Diego, CA, USA), *de novo* assembled with SOAPdenovo2 (Luo et al. 2012). The annotation was conducted using DOGMA (Wyman et al. 2004) and CpGAVAS (Liu et al. 2012). The tRNA was confirmed with tRNAscan-SE (Lowe and Eddy 1997).

The complete chloroplast genome sequence of *C. maculosa* was 152,518 bp and deposited in GenBank (MN228501). It consists of one large single-copy (LSC) (83,959 bp), one small single-copy (SSC) (18,487 bp), and two inverted repeat (IRs) regions (25,218 bp). The overall GC contents of cp genome were 43.1% and in the LSC, SSC, and IRs were 35.9, 31.4, and 37.7%, respectively.

The chloroplast genome contains 111 unique genes, including 80 coding genes, 4 rRNA genes, and 27 tRNA genes. Twenty of those genes were duplicated in IR regions (*ndhB, rpl2, rpl23, rps12, rps19, rps7, ycf15, ycf2, rrn4.5 rrn5, rrn16, rrn23, trnN-*GUU, *trnR-*ACG, *trnA-*UGC, *trnI-*GAU, *trnV*-GAC, *trnL-*CAA, and *trnM-*CAU) and 19 genes contained one or two introns.

The maximum likelihood phylogenetic tree was generated using RAxML (Stamatakis 2014) based on the complete chloroplast genome of *C. maculosa* and 16 other species from Asteraceae (8 Carduinae, 3 Centaureinae, 2 Carliniae, 2 Crepidinae, 1 Heliantheae, and 1 *Jacobea* as an outgroup). Most nodes in a phylogenetic tree were supported strongly. And the phylogenetic tree showed that *C. maculosa* was closely related to *Centaurea diffusa* (Figure 1). The complete chloroplast sequence of *C. maculosa* will provide a useful resource for molecular markers as determining invasive plants.

**Figure 1.**
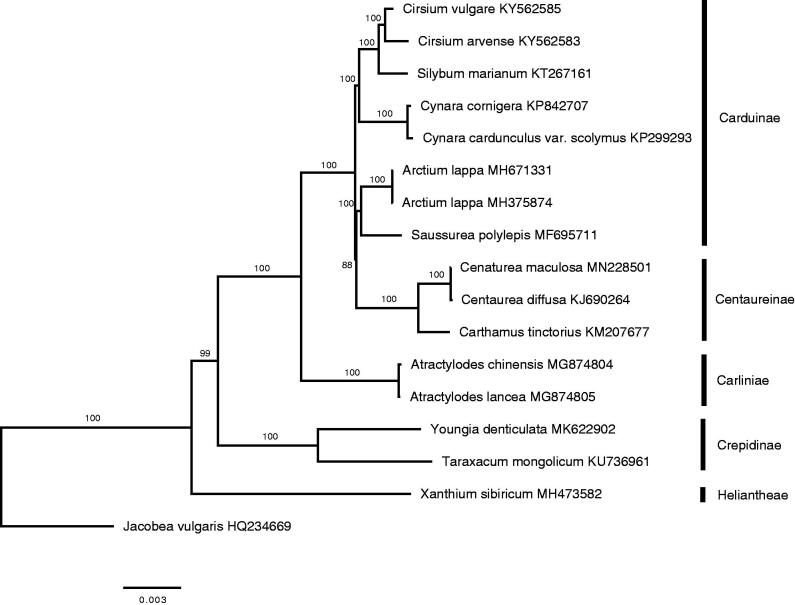
Maximum likelihood phylogenetic tree generated by RAxML based on complete chloroplast genome sequence of seventeen species from the family Asteraceae. The bootstrap value based on 1000 replicates is shown on branches.

## References

[CIT0001] Callaway RM, Newingham B, Zabinski CA, Mahall BE 2001 Compensatory growth and competitive ability of an invasive weed are enhanced by soil fungi and native neighbors. Ecol Lett. 4(5):429–433.

[CIT0002] Lacey CA, Lacey JR, Fay PK, Story JM, Zamora DL 1995 Controlling spotted knapweed on Montana rangelands. Bozeman: Montana State University Extension Service Publication; p. 311.

[CIT0003] Lowe TM, Eddy SR 1997 tRNAscan-SE: a program for improved detection of transfer RNA genes in genomic sequence. Nucleic Acids Res. 25(5):955–964. 9023104 10.1093/nar/25.5.955 PMC146525

[CIT0004] Luo R, Liu B, Xie Y, Li Z, Huang W, Yuan J, He G, Chen Y, Pan Q, Liu Y, et al. 2012 SOAPdenovo2: an empirically improved memory-efficient short-read de novo assembler. GigaSci. 1(1):18. 10.1186/2047-217X-1-18 PMC3626529 23587118

[CIT0005] Liu C, Shi L, Zhu Y, Chen H, Zhang J, Lin X, Guan X 2012 CpGAVAS, an integrated web server for the annotation, visualization, analysis, and GenBank submission of completely sequenced chloroplast genome sequences. BMC Genom. 13(1):715. 10.1186/1471-2164-13-715 PMC3543216 23256920

[CIT0006] Stamatakis A 2014 RAxML version 8: a tool for phylogenetic analysis and post-analysis of large phylogenies. Bioinformatics. 30(9):1312–1313. 24451623 10.1093/bioinformatics/btu033 PMC3998144

[CIT0007] Wyman SK, Jansen RK, Boore JL 2004 Automatic annotation of organellar genomes with DOGMA. Bioinformatics. 20(17):3252–3255. 15180927 10.1093/bioinformatics/bth352

